# Analysis of episiotomy incidence and risk factors in vaginal deliveries: a single-center

**DOI:** 10.1016/j.xagr.2024.100371

**Published:** 2024-07-17

**Authors:** Suskhan Djusad, Intan Indah Permatasari, Annisa Futihandayani, Puti Shahnaz, Daniel Hadiwinata, Hana Fathia Herianti

**Affiliations:** Department of Obstetrics and Gynecology, Faculty of Medicine Universitas Indonesia, Dr. Cipto Mangunkusumo National Central Public Hospital, Jakarta, Indonesia

**Keywords:** Episiotomy, risk, delivery, labor

## Abstract

**Introduction:**

Episiotomy is a surgical procedure involving the enlargement of the posterior vagina to facilitate the delivery of the baby. This study aims to further investigate the associated risk factors for episiotomy and the specific indications for its use in spontaneous labor.

**Methodology:**

This institutional-based cross-sectional study was conducted among 349 vaginal births with a ratio of 1:4 from January 2020 to December 2020. We recruited study participants using consecutive sampling techniques. The sample size was calculated with a hypothesis test for two population proportions (one-sided test formula). Adjusted odds ratio with the corresponding 95% confidence interval was used to declare the significance of variables.

**Results:**

In our multivariate analysis, it was found that pregnant women who underwent instrumental delivery (*P*-value=.00; OR=25.63; 95% CI: 5.76–114.0) and those with fetal birth weight >3,000 grams (*P*-value=.00; OR=11.31; 95% CI: 3.96–32.32) had the highest risk of undergoing an episiotomy. Subsequently, the duration of the second stage of labor >30 minutes (*P*-value=.049; OR=16.34; 95% CI: 1.01–264.48) was associated with a slightly increased risk of episiotomy. Fetal head circumference >34 cm was not found to be risk of an increased risk of episiotomy in this study. However, pregnant women aged >30 years (*P*-value=.049; OR=0.306; 95% CI: 0.94–0.99) showed a reduced risk of episiotomy.

**Conclusion:**

The prevalence of episiotomy practice in this study exceeds the recommended threshold set by the World Health Organization (WHO). Instrumental delivery, high birth weight, and prolonged second-stage labor emerged as significant factors influencing episiotomy practice. Hence, further interventions are warranted to mitigate the prevalence of episiotomy practice.

**AJOG Global Reports at a Glance**

**Why was this study conducted?**

Episiotomy, a common surgical procedure during vaginal delivery, is performed for various indications such as fetal position and macrosomia, despite global recommendations against routine use.

**Key findings**

A study at Cipto Mangunkusumo National General Hospital revealed a 20% episiotomy rate, exceeding WHO recommendations, with significant associations found between episiotomy and factors like instrumental delivery and prolonged labor duration.

**What does this add to what is known?**

The findings of this study highlight the need for personalized decision-making regarding episiotomy and emphasize the importance of reducing unnecessary interventions in childbirth.

## Introduction

An episiotomy is a second-stage vaginal delivery incision made in the perineum and posterior vaginal wall to widen the vaginal aperture and facilitate the vaginal birth process.^1^ Despite being one of the most common surgical procedures performed worldwide, the choice to perform an episiotomy is still based on several factors and indications, including the baby's position with persistent occipital presentation, shoulder dystocia, breech delivery, fetal macrosomia, and assisted vaginal birth using vacuum or forceps.^1^^,^^2^ One of the advantages of the episiotomy method is that there is a reduced chance of perineal tears, which means the incision will heal more quickly.^1^ The World Health Organization (WHO) advises against routine episiotomy, stating that the incidence should not go above 10% even though it is widely used.^3^ According to a Cochrane Library comprehensive review, compared to regular episiotomy, selective episiotomy performed during spontaneous labor without the use of equipment led to 30% fewer severe vaginal traumas. As a result, there has been doubt about the routine use of episiotomies due to research that suggests the dangers may not always outweigh the benefits.^4^^,^^5^

Considering the risk of unnecessary episiotomy, the Federation of Obstetrics and Gynecology (FIGO) in 2017 limited this procedure by considering it only in a few situations such as the vaginal birth has a risk of perineal laceration or the patient has an urgency of delivering the fetus.^6^ The Royal College of Obstetricians and Gynecologists (RCOG) also considers mediolateral episiotomy in operative vaginal delivery.^7^ 10 decades ago prevalence of episiotomy in Europe namely Sweden was (9.7%) and the United Kingdom was (12%–15%). Furthermore, the prevalence of episiotomy in developed countries still exceeds the WHO recommendation.^8^ In 2022, episiotomy in America decreased drastically from 67% in 2012 to 4.6%.^9^ In France, the prevalence of episiotomy decreased from around 26.7% in 2007 to 19.9% in 2014.^10^ Meanwhile, developing countries like Turkey, still have a high rate of episiotomy in childbirth up to 52% with 93.3% of them in primiparous women and 30.2% in multiparous women.^11^ Other developing countries have also shown a liberal use of episiotomy by a very high rate of episiotomy in all vaginal delivery, with rates as high as 63.4% in India, 66% in Oman, 94.5% in Cambodia, and the highest in Taiwan with 100%.^12^

Data about episiotomy's prevalence in Indonesia is still limited. A study in Indonesia, in 2022, has shown that 33% of pregnant women with vaginal delivery had episiotomy.^13^ Meanwhile, a study in 2019–2021 at Mohammad Hoesin General Hospital showed that the incidence rate was much higher up to 73.3%.^14^ The data from 2022 showed improvement from the one established in 2019–2021, however, it still exceeds the WHO recommendation. In this study, the author aims to not only analyze the incidence of episiotomy in Cipto Mangunkusumo National General Hospital but also to enable better decision-making and encourage a decrease in rates of episiotomy by WHO guidelines.

## Methodology

The study design was an analytical, observational, case-control with dividing samples into two groups (case group and control). Sample selection was conducted at Dr. Cipto Mangunkusumo National Hospital between January and December 2020. A consecutive sampling was acquired, according to the inclusion and exclusion criteria. All the pregnant women who gave birth vaginally and voluntarily agreed to sign an informed consent were included in the study. Exclusion criteria were pregnant women with intrauterine infection or infection of the birth canal, impaired neurological function, and infants with congenital abnormalities such as anencephaly. This research study has obtained ethical approval from the Research Ethics Committee of the Faculty of Medicine, University of Indonesia (No KET/1759/UN2.F1/ETIK/PPM.00.02/2023). Women who experienced an episiotomy during labor were cases, while those who did not undergo an episiotomy were designated as the control group. Sample calculation was analyzed using a *Hypothesis test for two population proportions* (*one-sided test*) and obtained 35 cases so that the total minimum sample was 70. The dependent variable was episiotomy (mediolateral or midline). In contrast, the independent variables included the subject's demographic data (age and parity), fetal characteristics (fetal presentation, birth weight, and head circumference), type of vaginal delivery (spontaneous or instrumental), and duration of labor in Stage II. Data was analyzed statistically using *SPSS 22 ver.* To show the relation between episiotomy and independent variable (predictors), the *chi-square* test and *Fisher's test* were performed with a significance value >0.05. The odds ratio (OR) and 95% confidence interval (95% CI) were also calculated as a result of the multivariate logistic regression analysis.

## Results

During the study period, a total of 349 women with vaginal delivery were qualified for the inclusion criteria. The ratio of case to control was 1:4, resulting in 34 cases that underwent episiotomy and 136 in the control group, bringing the total to 170 samples. Because episiotomy is rarely performed without indications, we found very few cases of mediolateral episiotomy. Therefore, out of 349 patients, we only used 170 patients. The characteristics of the 170 samples are shown in Table 1 with a 20% rate of episiotomy. The age range of participants was 14 to 45 years. One hundred-seventeen (68.8%) of pregnant women were primiparous compared to multiparous (31.2%), and the majority (88.8%) of them were delivered spontaneously without an instrument. Based on fetal presentation, one hundred fifty-eight (92.9%) participants presented with vertex presentation and twelve (7.1%) with breech presentation. The average birth weight was mostly 2665 grams with 31 cm of head circumferences.

**Table 1:** Characteristics of samples

Characteristics	*N*=170 (%)
Age (years)	28 (14–45)
Parity	
Primipara	117 (68.8%)
Multipara	53 (31.2%)
Fetal presentation	
Vertex	158 (92.9%)
Breech	12 (7.1%)
Birth weight (gram)	2665 (658–3875)
Head circumferences (cm)	31 (19–36)
Vaginal delivery	
Spontan	151 (88.8%)
Instrumental	19 (11.2%)
Episiotomy	
Not-performed	136 (80%)
Mediolateral	34 (20%)

Bivariate analysis of maternal factors showed that subjects who gave birth vaginally with either vacuum or forceps had a significant effect on episiotomy practices (*P*-value=.00; OR=26.05; 95% CI: 7.82–86.77). The duration of second-stage labor is also associated with episiotomy (*P*-value=.006; OR=18.0; 95% CI: 1.94–1666.85). Some fetal factors directly relate to the indication of episiotomy during labor, such as birth weight above 3,000 grams (*P*-value=.00; OR=10.65; 95% CI: 4.52–25.07), and fetal head circumference above 34 cm (*P*-value=.01; OR=3.87; 95% CI: 1.39–10.76). The results can be seen in Table 2.

**Table 2:** Risk factors associated with episiotomy

Variables	Cases (*n*=34)	Control (*n*=36)	*P*	OR (IK 95%)
Age			.102	0.51 (0.22–1.15)
>30 years	10 (29.5%)	61 (44.9%)		
<30 years	24 (70.5%)	75 (55.1%)		
Parity			.282	0.62 (0.26–1.48)
Primipara	26 (76.5%)	91 (66.9%)		
Multipara	8 (23.5%)	45 (33.1%)		
Fetal presentation			1.00^	0.78 (0.16–3.77)
Vertex	32 (94.1%)	126 (92.6%)		
Breech	2 (5.9%)	10 (7.4%)		
Birth weight (gram)			.00*	10.65 (4.52–25.07)
>3.000	24 (70.5%)	25 (18.4%)		
<3.000	10 (29.5%)	111 (81.6%)		
Head circumferences (cm)			**.011**⁎^	3.87 (1.39–10.76)
>34	8 (23.5%)	10 (7.4%)		
<34	26 (76.5%)	126 (92.6%)		
Vaginal delivery			**.00**⁎^	26.05 (7.82–86.77)
Spontan	19 (55.9%)	132 (97.1%)		
Instrumental	15 (44.1%)	4 (2.9%)		
Duration of the second stage of labor (minute)			**.006**⁎^	18.00 (1.94–1666.85)
>30 min	4 (11.8%)	1 (0.8%)		
<30 min	30 (88.2%)	135 (99.2%)		

Bold values are statistically significant with p value < 0.05.

Chi-square's test.

^ Fisher's exact test

⁎ Statistically significant with *P*-value<.05.

When the multivariate analysis was generated with “LR backward” regression, women who performed instrumental delivery (*P*-value=.00; OR=25.63; 95% CI: 5.76–114.0) and had a baby with birthweight >3,000 grams (*P*-value=.00; OR=11.31; 95% CI: 3.96–32.32) were more likely to have the highest risk of episiotomy. Following that, the duration of second-stage labor >30 minutes (*P*-value=.049; OR=16.34; 95% CI: 1.01–264.48) and maternal age >30 years (*P*-value=.049; OR=0.306; 95% CI: 0.94–0.99) decreased risk of episiotomy. Meanwhile, fetal head circumference >34 cm was not a factor associated with the risk of episiotomy in this study (Table 3).

**Table 3:** Multivariate analysis of the risk factors associated with episiotomy

Variables	B	SE	Cases (*n*=34)	Control (*n*=136)	*P*	OR (IK 95%)
Konstanta	-8.73	1.86				
Age	-1.18	0.60			**.049**	0.306 (0.94–0.99)
>30 years			10 (14.1%)	61 (85.9%)		
<30 years			24 (24.2%)	75 (75.8%)		
Birth weight (gram)	2.42	0.53			**.00**	11.31 (3.96–32.32)
>3.000			24 (49%)	25 (51%)		
<3.000			10 (8.3%)	111 (91.7%)		
Head circumferences (cm)	0.83	0.67			.21	2.30 (0.61–8.64)
>34			8 (44.4%)	10 (55.6%)		
<34			26 (17.1%)	126 (82.9%)		
Vaginal delivery	3.24	0.76			**.00**	25.63 (5.76–114.00)
Spontan			19 (12.6%)	132 (87.4%)		
Instrumental			15 (78.9%)	4 (21.1%)		
Duration of the second stage of labor (minute)	2.79	1.42			**.049**	16.34 (1.01–264.48)
>30 min			4 (80%)	1 (20%)		
<30 min			30 (18.2%)	135 (81.8%)		

Bold values are statistically significant with p value < 0.05.

## Discussion

The prevalence of episiotomy in our study was approximately 20%, surpassing the recommended limit set by the World Health Organization (WHO) in 2018, which is 10% for all normal deliveries.^3^ These findings highlight the common occurrence of episiotomy at Cipto Mangunkusumo Public Hospital, particularly among primiparous pregnant women. It's worth noting that our result, although higher than the WHO guideline, is lower than the prevalence reported in a study conducted at the Palembang Maternity Clinic, where it reached 30.8%. It is important to consider that this disparity may be attributed to differences in sample size and research methods, particularly the cross-sectional design employed in the previous study.^15^ Furthermore, comparing our results to a study at Haramaya University, Ethiopia, conducted in 2022, where the prevalence of episiotomy was found to be higher at 43.4% (95% CI: 38.7–48.9), adds a broader perspective.^5^ This variation emphasizes the need to recognize regional differences and contextual factors influencing episiotomy rates. The contribution of our study, along with previous research, lies in providing new insights for clinical practice, especially regarding complex risk factors closely linked to the prevalence rate of episiotomy.^15^ These findings can inform healthcare professionals and policymakers in refining obstetric practices to ensure optimal maternal outcomes.

Our study indicates that certain labor factors significantly influencing the incidence of episiotomy include the duration of the second stage and the use of instruments during delivery. Notably, our findings align with a cohort study by Zang et al. (2018) involving 3721 pregnant women subjects, which highlighted that a prolonged second stage (>1 hour for primiparas and >30 minutes for multiparas) substantially increased the likelihood of episiotomy.^16^ Specifically, primiparas faced a twofold chance of undergoing episiotomy in such cases. However, our results differ from a study conducted in Finland, where a cutoff time of >60 minutes for the second stage revealed a 2.9 times higher risk for episiotomy in multiparas and a 1.3 times higher risk in primiparas. Intriguingly, the Finish study also emphasized that the designation of a "protracted second stage" was not a definitive indicator of the necessity of episiotomy.^17^ Furthermore, some research subjects expressed anxiety about the extended waiting period during the second stage of labor. In the context of labor with a prolonged second stage, the decision to perform episiotomy is often made for pregnant women with a history of previous diseases such as hypertension. This practice is believed to mitigate the risk of postpartum complications. This nuanced approach highlights the need for personalized considerations in making decisions about episiotomy, taking into account both the duration of the second stage and the individual health history of the pregnant woman.^17^

The findings of our study align with those of a cross-sectional study conducted by Woretaw et al. (2021) involving 410 subjects who underwent normal delivery with instrumental assistance, showed a threefold higher risk of episiotomy.^18^ Additionally, instrumental delivery emerged as a significant risk factor for episiotomy in a study by Braga GC, presenting a twelvefold higher risk.^19^ Other research studies have demonstrated that episiotomy procedures were frequently performed in cases involving cephalic hematoma and grade 3–4 perineal lacerations during instrumental vaginal deliveries. Furthermore, the use of epidural analgesics contributes to instrumental deliveries. The impact of anesthesia diminishes the effectiveness of the mother's pushing efforts, leading to inadequate pushing efforts that may culminate in the necessity for "tool assistance" or episiotomy.^20^ It's noteworthy to mention that the combination of instrumental delivery and episiotomy is cautioned against by several previous studies due to the elevated incidence of Obstetric Anal Sphincter Injuries (OASIS). These collective findings underscore the importance of careful consideration and cautious approaches in instances involving instrumental deliveries to minimize the risk of complications, particularly those associated with episiotomy and subsequent adverse outcomes like OASIS.^20^

In bivariate analysis (Table 2), fetal factors such as birth weight and head circumference showed a significant relationship with episiotomy. However, the multivariate analysis, as presented in Table 3, indicated that only constant birth weight maintained a significant association. Specifically, in our study, a birth weight exceeding 3 kg was associated with episiotomy, with approximately 49% of the case group population undergoing the procedure. This finding is consistent with existing research that establishes a correlation between birth weight and the likelihood of episiotomy. Studies suggest that for every additional 100 grams of weight, there is an increase in the risk of episiotomy by approximately 5.4%–6.1% in primiparas. Furthermore, in various studies, a body weight equal to or exceeding 4000 grams has been identified as a predictive factor for episiotomy.^16^^,^^21^ The heavier body weight is often linked to a larger fetal head, posing challenges for the fetus to pass through the perineal area, thus necessitating episiotomy to widen the vaginal orifice. However, it's noteworthy that a body weight of ≥3800 grams does not exhibit a close correlation with episiotomy. It's essential to acknowledge that the relationship between birth weight, fetal macrosomia, and episiotomy remains a topic of controversy in existing literature. Further research may be necessary to gain a more comprehensive understanding of these complex interactions.^21^^,^^22^

Research in Brazil has identified that primiparous pregnant women consistently face the highest risk of undergoing an episiotomy, followed by instrumental delivery, and the presence of a doctor during the birthing process.^19^ This aligns with previous systematic review studies, which concluded that episiotomy in primiparas (as opposed to multiparas) serves as a protective factor against anal sphincter lesions.^23^ The benefits of episiotomy in primiparas include accelerating the healing of surgical wounds compared to perineal lacerations without episiotomy, along with reductions in pain, bleeding, infection, urinary incontinence, and the risk of rectal fistula. Despite primiparas being globally recognized as the maternal group most at risk for episiotomy, your study yielded different results. Among the 177 (68.8%) primiparous subjects, 22.2% were in the group that underwent episiotomy. However, the results of bivariate analysis did not reveal a significant relationship between the parity status of pregnant women and the incidence of episiotomy (*P*-value: 0.282). These findings contribute valuable insights, emphasizing the need for nuanced considerations and understanding regional variations in obstetric practices.

The findings of this study align with research by Modi A, reinforcing the notion that routine episiotomy may not offer substantial benefits for primiparous pregnant women. In Modi A's prospective non-randomized case-control study, outcomes were compared between a group subjected to routine episiotomy (control group) and a group undergoing selective episiotomy (case group). The selective episiotomy group exhibited fewer cases of perineal pain three days post-delivery compared to the control group, indicating that selectively performed episiotomy may lead to better maternal clinical outcomes. This observation supports the recommendation against routine episiotomy in spontaneous vaginal births, as endorsed by the World Health Organization (WHO). These collective findings underscore the importance of adopting a selective approach to episiotomy, considering individualized maternal factors and clinical circumstances to optimize outcomes and minimize unnecessary interventions.^3^^,^^24^^,^^25^

The limitations of our study lie in the small sample size and population, which is only limited to the scope of one hospital. We have not explored the complications of episiotomy further. Our suggestion, future research should compare the effectiveness of routine episiotomy with selective episiotomy. In this study, the rate of episiotomy still did not reach the recommended target rate from WHO which is below 10%. Spontaneous vaginal delivery with vacuum or forceps and prolonged duration of second-stage labor are the main factors that can be prevented for reducing the proportion of episiotomy in Indonesia. On the other hand, maternal age >30 years and fetal birth weighing >3000 grams need to be carefully considered for the possibility of an episiotomy. Therefore, doctors and midwives should be wiser in determining indications for an episiotomy based on the risk factors that influence it.

## Condensation

A general hospital in Indonesia found a 20% prevalence of episiotomy primarily among primiparous women, with factors such as instrumental delivery and prolonged second-stage labor significantly influencing its incidence, underscoring the importance of personalized decision-making to reduce unnecessary episiotomies.

## CRediT authorship contribution statement

**Suskhan Djusad:** Validation, Supervision, Resources, Project administration, Methodology, Investigation, Funding acquisition, Formal analysis, Data curation, Conceptualization. **Intan Indah Permatasari:** Writing – original draft, Software, Resources, Data curation. **Annisa Futihandayani:** Writing – original draft, Software, Conceptualization. **Puti Shahnaz:** Writing – review & editing, Writing – original draft, Visualization, Validation. **Daniel Hadiwinata:** Visualization, Software, Formal analysis. **Hana Fathia Herianti:** Writing – review & editing, Writing – original draft, Visualization, Validation.
